# Birdshot retinochoroiditis in Brazil: a multicenter review of 40 patients

**DOI:** 10.1186/s40942-021-00353-1

**Published:** 2022-01-07

**Authors:** Maria Luisa Gois da Fonsêca, Raul N. G. Vianna, Anna C. H. Rocha, Antonio M. B. Casella, Arnaldo Cialdini, Cristina Muccioli, Daniela S. da Costa, Daniel R. Lucena, Daniel V. Vasconcelos-Santos, Eduardo Morizot, Elaine Castro, Ever E. C. Rodriguez, Felipe T. Diligenti, Fernanda B. O. Porto, Heloisa Nascimento, Joyce H. Yanamoto, Juliana L. Oréfice, Lilia R. P. Muralha, Luciana B. Carneiro, Luciana P. S. Finamor, Maria A. M. Frazão, Mario Motta, Mario J. Nobrega, Moyses E. Zajdenweber, Remo T. Moraes, Rodrigo L. Meirelles, Sidney R. Lemos, Wilton Feitosa de Araújo

**Affiliations:** 1 Retina and Vitreous Service, Federal Fluminense University (UFF), Marques do Parana Avenue 303 Centro, Niterói, RJ 24033900 Brazil; 2 Retina and Vitreous Service, Federal University of Minas Gerais (UFMG), Belo Horizonte, MG Brazil; 3 Retina and Vitreous Service, Londrina State University (UEL), Londrina, PR Brazil; 4 Retina and Vitreous Service, Brazilian Center for Eye Surgery (CBCO), Goiânia, GO Brazil; 5 Retina and Vitreous Service, Federal University of São Paulo (UNIFESP), São Paulo, SP Brazil; 6 Retina and Vitreous Service, Federal Hospital of State Servers (SERVIDORES), Rio de Janeiro, RJ Brazil; 7 Retina and Vitreous Service, Escola Cearence de Oftalmologia, Fortaleza, CE Brazil; 8 Retina and Vitreous Service, Botafogo Polyclinic, Rio de Janeiro, RJ Brazil; 9 Retina and Vitreous Service, Federal University of Rio Grande do Sul (UFRGS), Porto Alegre, RS Brazil; 10 INRET-Clinica e Centro de Pesquisa, Belo Horizonte, MG Brazil; 11 Ophthalmology Department, Hospital das Clínicas, HCFMUSP (USP), São Paulo, SP Brazil; 12 Centro Brasileiro de Ciências Visuais, Belo Horizonte, MG Brazil; 13 Hospital de Olhos de Niterói, Niterói, RJ Brazil; 14 Fundação de Olhos de Góias (FUBOG), Goiânia, GO Brazil; 15 Department of Ophthalmology, “Santa Casa”of São Paulo, São Paulo, SP Brazil; 16 Retina and Vitreous Service, Federal University of the State of the Rio de Janeiro (UNIRIO), Rio de Janeiro, RJ Brazil; 17 Retina and Vitreous Service, Hospital de Olhos Sadalla Amin Ghanem, Joinville, SC Brazil; 18 Brazilian Institute of Ophthalmology (IBOL), Rio de Janeiro, RJ Brazil; 19 Retina and Vitreous Service, Santa Casa de Belo Horizonte, Belo Horizonte, MG Brazil

**Keywords:** Birdshot retinochoroiditis, Retina, Uveitis

## Abstract

**Background:**

Birdshot retinochoroiditis (BRC) is a rare and chronic bilateral uveitis mostly found in Caucasians. As few data are available about the clinical course of BRC in Hispanic patients, we aimed to report the clinical findings and the evolution of BRC in Brazilian patients.

**Methods:**

This retrospective cohort multicenter nationwide study was performed by analyzing the records of patients with BRC diagnoses from Brazilian ophthalmological centers from April 1995 to May 2020.

**Results:**

Forty patients (80 eyes) with a diagnosis of BRC were evaluated. The mean age was 53 years, and there was no sex predominance. All tested patients (34/40) were positive for HLA-A29. The diagnosis of BRC was made following the Levinson et al. criteria, and all ancillary tests were performed to exclude differential diagnoses. Clinical signs and symptoms, such as complications and treatment, were described.

**Conclusions:**

BRC evolution in Brazilian patients seems to have some peculiarities that diverge from the published literature available about Caucasians, as AS inflammation is higher in this population.

## Introduction

Birdshot retinochoroiditis (BRC) is a rare form of bilateral, chronic, posterior uveitis with distinct fundus lesions. The origin of its name is derived from the characteristic fundoscopic lesions that resemble birdshots by a shotgun. Patients affected by BRC usually have good visual acuity (VA) with minor complaints at the onset of the disease; however, as the disease advances, it may cause vision loss. Common causes of vision loss are refractory central macular edema (CME), choroidal and retinal neovascularization, optic atrophy, cellophane maculopathy, macular scarring, and glaucoma [1–3]. A noteworthy feature of BRC is the fact that it is firmly connected to HLA-A29, suggesting a strong genetic predisposition [4].

BRC was first reported by Ryan and Maumenee in 1980. Their patients had common features associated with typical fundus lesions located at the RPE area or deeper [5]. In 1982, Gass suggested the name “vitiliginous chorioretinitis” for the illness, particularly because of the characteristics of the lesions. He described a series of cases with 11 patients and reinforced the choroidal position of the lesions [6]. At the present moment, the most accepted name is birdshot retinochoroiditis. However, Herbort and colleagues recently suggested modifying the name to “HLA-A 29 uveitis” due to its close connection to this malady and because the characteristic fundus lesions may not be present at the initial stage of BRC [4].

The close association between HLA-A29 and BRC was first reported by Nussenblat et al*.*, who described 80% HLA-A29 positivity in BRC patients compared to controls (7.4%) [7]. The total frequency of HLA-A29 worldwide is 4.5%; however, HLA-A29 is more commonly seen in Caucasians [8]. BRC seems to be a prototype of an HLA disease that is expressed similar to uveitis, with choroid and retina commitment [9]. Nevertheless, the fact that HLA-A29 is present in a considerable percentage of the Caucasian population but rarely manifests as BRC suggests that other genes may be involved in this process.

After an international conference, a consensus about BRC diagnostic criteria was published by Levinson et al. [10], and these criteria were used in this study.

The classification of race in Brazil is challenging and controversial because of the high miscegenation in the history of the country. Most likely, Caucasian ancestry is not precise, as the color of the skin is not an accurate parameter to define ancestry in a highly miscegenated population such as the Brazilian population [11–13]. A Caucasian ancestry in patients classified as “white” can be suggested; a genetic study, however, would be necessary to corroborate this classification. BRC is extremely rare among other ethnicities. In fact, very few reports about the BRC clinical course in the Hispanic population can be found in the literature (Table 1) [14–17]. In this article, we report the clinical findings and the evolution of BRC among Brazilian patients.

**Table 1:** Table demonstrating previous studies with Hispanic population

Study	Year	Sample size	HLA-A29	Age	AS inflammation	Clinical features	Complications
Gasch et al.	1999	1 (59patients)	Positive	Not available	Not available	Not available	Not available
Torres- Soriano et al.	2008	1	Not available	45	Negative	Low vision	Cataracts
Rodríguez-García et al.	2012	6	Positive (5/6)	Mean 46.5	Positive (4/6)	Blurred vision, myodopsies	Cataracts (7), glaucoma (4), CNV (1), CME (2);CM (1)
Baddar et al.	2016	1	Positive	62	Positive	Floaters, low vision, nyctalopia	–

*AS *anterior segment, *CNV* choroidal neovascularization, *CME *central macular edema, *CM* cellophane maculopathy

## Patients and methods

### Design

In this multicenter retrospective observational cohort study, patient records from April 1995 to May 2020 with a clinical diagnosis of BRC from the 19 largest Brazilian ophthalmological centers were analyzed. Information on patient demographics, ocular features, investigations, management, and treatments were collected from medical records. The study was conducted in accordance with the guidelines of the 1964 Declaration of Helsinki.

#### Diagnostic protocol

The symptoms and signs of the first evaluation of each patient were considered. Only the final BCVA and complications were evaluated at the last visit. Considering the rarity of the disease, to be able to obtain an adequate sample size, patients were evaluated in different years for different specialists; however, some of them had been lost to follow-up through the years, with only the medical records available to provide information on ancillary exams.

The study adopted the following criteria proposed by Levinson et al. in 2006 for the diagnosis of BRC [10]:

- Inclusion criteria: (i) bilateral disease, (ii) the presence of at least three peripapillary “birdshot lesions” inferior or nasal to the optic disk in one eye, (iii) low-grade anterior segment (AS) intraocular inflammation, and (iv) low-grade vitreous inflammatory reaction.
- Supportive findings: (i) HLA-A29 positivity, (ii) retinal vasculitis, and (iii) cystoid macular edema (CME).
- Exclusion criteria: (i) keratic precipitates, (ii) posterior synechiae, and (iii) the presence of infectious, neoplastic, or other inflammatory diseases that can cause multifocal choroidal lesions.

With time, AS intraocular inflammation was proven not to be prevalent among BRC patients, as will be discussed in the text. Thus, if AS intraocular inflammation was not present, the diagnosis of BRC could still be made. Ancillary tests associated with the supportive findings were employed in this sense. All these patients underwent IGRA (interferon gamma release assay), PPD (purified protein derivative), VDRL (venereal disease research laboratory), FTA-ABS (fluorescent treponemal antibody absorption), chest X-ray, and ACE (angiotensin converting enzyme) serum level analyses, with negative results. The differential diagnosis with other inflammatory diseases, such as Vogt–Koyanagi–Harada disease, intraocular lymphoma, acute posterior multifocal placoid pigment epitheliopathy, punctate inner choroidopathy, multifocal evanescent white-dot syndrome, pars planitis syndrome, posterior scleritis, sympathetic ophthalmia, and multifocal choroiditis, was made based on the finding of typical retinal birdshot lesions and the absence of typical features of these other diseases in these patients.

### Data collection

Information on demographics, comorbidities, family history, symptoms, clinical findings, ancillary exams, complications, and treatment were extracted from the medical records of the patients, sent to the data center at the Department of Ophthalmology of the University Hospital Antonio Pedro of Federal Fluminense University, and collected by the first author (MLGF). Ophthalmological signs and complications such as vitritis, vasculitis, disc edema, CME, retinal and choroidal neovascularization, optic atrophy, and epiretinal membrane were evaluated through fluorescein angiography (FA) and optical coherence tomography (OCT).

The individuals were divided by the color of their skin into white and nonwhite categories. Individuals with “black” and “brown” skin colors were condensed into the nonwhite category. The melanin reaction to the sun according to Fitzpatrick's classification, based on patient report, was noted [18].

### Statistics

The descriptive statistics used were the mean, range, and standard deviation. The normality of data distribution was tested by the Shapiro–Wilk test, and the chi-squared test was used for comparisons between the white and nonwhite categories. The analyses were performed with the Statistical Package for the Social Sciences version (IBM SPSS Statistics, Chicago, IL) and ASP (JASP Team (2020) JASP (Version 0.13.1) [Computer software], University of Amsterdam).

## Results

The mean follow-up was 61.5 ± 76.85 months (range 3–300 months).

### Demographics

Forty patients (80 eyes) diagnosed with BRC were evaluated. The mean age was 53 ± 14 years (range 18–86 years), with no sex predominance. Seventy-five percent [30] were classified as “white”, and 25% [10] were classified as “nonwhite”. All patients were native Brazilians, and no information about ancestry was available. The mean age of patients in the “white” category was 55.23 ± 14.6, and that of patients in the “nonwhite” category was 45.13 ± 6.6.

### Clinical features

The initial symptoms included decreased vision/blurred vision in 86.5% (32/37), floaters in 38% (14/37), photopsia in 22% (8/37), nyctalopia in 11% (4/37), and metamorphopsia in 2.7% (1/37) of patients, with more than one symptom in 49% (18/37) of patients. Data for three patients, that is, 7.5% (3/40), were missing.

### Ocular signs

Eighty percent (32/40) of patients had vasculitis, 62.5% (25/40) had disc edema, 60% (24/40) had CME, and 40% (16/40) had AS inflammation. These data are by patient, regardless of whether one or both eyes were affected.

Only four patients had an initial BCVA of 20/20 in both eyes, with the majority of eyes presenting with BCVA between 20/25 and 20/60. The percentages of individuals whose BCVA was maintained, worsened, and improved were almost the same, at 28.75%, 31.25%, and 30%, respectively.

The only variable that for which a near statistical significance (p = 0.07) was observed when comparing the “white” × “nonwhite” categories was that the percentage of patients in the “white” category with diminished vision was lower than the percentages of patients who had improved or sustained vision when comparing the vision at the first and last examination. This finding was not observed in the “nonwhite” category (Table 2).

**Table 2:** Comparison white × nonwhite improvement in VA

White					
		Variable	Counts	Proportion	p-value
RE	Improved/maintained BCVA		8	0.308	0.076
	Reduced BCVA		18	0.602	0.076
LE	Improved/maintained BCVA		8	0.308	0.076
	Reduced BCVA		18	0.602	0.076

*OBS* these information was not available for four patients; *VA* visual acuity; *BCVA* best corrected visual acuity; *RE *right eye; *LE* left eye

### Ancillary tests

HLA-A29 was positive in all patients who underwent HLA-A29 testing, but this testing was not performed in six patients for economic reasons.

Indocyanine green angiography (ICGA) was performed on 35% (14/40) of patients, all of whom showed hypofluorescent dark dots (HDDs).

Electroretinography (ERG) was also performed at presentation on 42.5% (17/40) of patients; 41.2% (7/17) had unspecified subnormal electroretinograms, 29.4% (5/17) had normal electroretinograms, 23.17% (4/17) had diminished b-waves and 12% (2/17) had diminished oscillatory potentials.

Visual field (VF) tests were performed at presentation on 40% (16/40) of patients, of whom 31.25% (5/16) had normal results, 25% (4/16) had a diffuse decrease in sensitivity, 25% (4/16) had visual field constriction, 12.5% (2/16) had enlarged blind spots, 12.5% (2/16) had a central scotoma, and 6.25% (1/16) had a loss of the superior field.

OCT was performed at presentation in nine patients. The most common finding was atrophied EPR foci (44, 4%; 4/9), followed by macular atrophy [2], a loss of the ellipsoid zone in the macular area [1], macular cysts [1], and macular holes [1].

Data were collected from twenty-nine retinal specialists. Therefore, different models and brands of equipment for FA, OCT, VF tests, and ERG were used in this study.

### Complications

Cataracts developed in 35% (14/40) of patients, epiretinal membrane in 25% (10/40), glaucoma in 15% (6/40), optic atrophy in 15% (6/40), and retinal and choroidal neovascularization in 5% (2/40). These data are by patient, regardless of whether one or both eyes were affected.

Table 3 presents a comparison of our data with those found in the literature.

**Table 3:** Comparison with the literature in Caucasians

Study	Year	Age	Macular edema (%)	Optic disc edema (%)	Retinal NV (%)	Choroidal NV (%)	Optic atrophy (%)	Epiretinal membrane (%)	Cataract (%)	Glaucoma (%)
Priem and Oosterhius	1988	52.5	NA	NA	7.4	6	3.5	10	NA	NA
Gasch et al.	1999	48.2 ± 9.9	37	14	1.7	2.6	0.9	6	NA	NA
Rothova et al.	2004	53	84	NA	NA	14	NA	NA	22	7
Shah et al.	2005	53 ± 9.4	50.5	23.9	3.2	5.9	8.5	8.1	NA	NA
Silpa-archa et al.	2016	51 ± 10	27	NA	NA	0	NA	30	97	NA
Our study	2021	53 ± 14	60	62.5	5	5	15	25	35	15

*NV* neovascularization; *NA* not available

### Treatment

Treatment was not received by 5% (2/40) of patients. Treatment information was not available in three patients. Of the patients who were treated, 97% (34/35) received systemic corticosteroids, 48.6% (17/35) azathioprine, 40% (14/35) cyclosporine, 11.4% (4/35) Ozurdex®, 11.4% (4/35) methotrexate, 8.3% (3/35) mycophenolate mofetil, and 8.6% (3/35) adalimumab. Systemic corticosteroids (prednisone) were used to treat 17.1% (6/35) of the patients. Two medications were used in 48.6% (17/35) of the patients; only in two of these were corticosteroids not used (the combinations were cyclosporine and azathioprine and azathioprine and mycophenolate mofetil). Three medications were used in 14.3% (5/35) of patients, four in 5.7% (2/35), and five in 8.6% (3/35). The most common combination, however, was corticosteroids along with azathioprine, as administered in 28.6% (10/35) of the patients.

## Discussion

The mean age of our patients was in accordance with that found in the literature. This study found a higher incidence of CME, optic disc edema, optic disk atrophy, and epiretinal membrane than those reported in the literature. Most of our patients complained about blurred or decreased vision at the first visit. In FA, typical BRC lesions show no signal or are relatively hypofluorescent. With the chronification of the disease, the lesions gain late hyperfluorescence by window defects [19, 20]. All of our patients had typical lesions. Gass described a prolonged arteriovenous time that was later proposed to be an exudative stage [6, 20, 21]. Based on these findings, we believe that the majority of our patients were in an active stage of the disease when they sought assistance, influencing the management and prognosis. Nevertheless, some of our patients were in a very advanced stage with a very low BCVA (< 20/400) at presentation. A comparison of our data with those in the literature can be seen in Tables 1 and 3.

AS inflammation is not a diagnostic criterion and is irrelevant to BRC. In the Priem and Oostherius series, only ten of 68 patients had mild flares and cells in the anterior chamber [2] Additionally, more recent studies using laser-flare cell photometry have shown minimal AS inflammation [22, 23] In the current study, 40% of the patients had AS inflammation, which is a higher prevalence than that previously described. We also attribute this finding to the fact that most patients are in the inflammatory and more symptomatic phase when looking for assistance.

Data from ethnicities other than Caucasian are very sparse in the literature. Table 1 summarizes the articles describing BRC in this group. When compared with our study, AS inflammation also had a higher prevalence among these other ethnicities in the literature, and cataracts and glaucoma were also common complications. Regarding this rarity, the first case describing BRC in Brazil was published in 1994 [24], and since then we have seen sparse literature. Also, it is worthy mentioned that a large series about uveitis in Brazil, with 1053 patients analyzed in 15 months, there were only 9 patients with white-dot syndromes, and BRC was not mentioned [25].

Patients classified as “white” were prevalent in our series. There is high variability in genes within wide cohorts of people belonging to the same specific ethnic group, making it impossible to make a more accurate classification of ethnicity [11–13]. Since BRC is more prevalent in Caucasians, perhaps a common ancestry could be suggested in these patients, but a genetic study would be necessary to corroborate this possibility.

The percentages of individuals whose BCVA was maintained, worsened, and improved were almost the same, at 28.75%, 31.25%, and 30%, respectively. This finding may reveal that treatment was unable to change the visual prognosis in BRC over a long period of time. In the study of Thorne et al., the 5-year cumulative incidence of a visual acuity of 20/200 or worse was 20%, similar to the finding of the study of Rothova et al. [3, 26], revealing a high percentage of visual loss despite treatment. However, the series of Rodríguez-García and colleagues suggested that long treatment with corticosteroids alone may be related to poor visual prognosis [16]. However, when the individuals were divided into “white” and “nonwhite” categories, in the “white” category, the percentage of individuals who had an improvement or maintained vision was higher than that of the individuals who had worsened vision (Table 2).

When assessing complications in our patients, cataracts were found to be the most common, and it could not be determined whether cataracts were a complication of the disease or of the treatment. Once the mean age of our patients was the same as that of patients with age-related cataracts, we could differentiate them by the characteristics of uveitis-complicated cataracts, which are caused by inflammation and treatment, mostly corticosteroids, with the most common type being posterior subcapsular uveitis-complicated cataracts [27, 28] Glaucoma is the second most prevalent complication, and its occurrence was similar to that described in the literature. One important consideration was that all patients who underwent intravitreal triamcinolone (four patients) developed glaucoma, and three developed cataracts. The other two patients who developed glaucoma received oral corticosteroids. Only two patients received only Ozurdex as an intravitreal corticosteroid, and the data for cataracts and glaucoma in these patients were not uniform; the same occurred in the two patients who were treated with subtenon dexamethasone. Optic atrophy along with retinal and RPE atrophy occurred in more patients than described in the literature, which is attributed to the small sample size and the stage of the disease at the first examination.

Less than half of our patients underwent ERG, and these patients did so only early after presentation. The most common findings were subnormal electroretinograms and diminished b-waves, showing a more advanced stage of the disease [29, 30] Additionally, two patients had diminished oscillatory potentials, which is an alteration associated with uveitis suggested as a marker for monitoring BRC patients [31]. None of our patients had information about the 30 Hz flicker implicit time, which has been related to a better visual prognosis and used in guiding treatment in BRC patients [29, 30, 32, 33].

VF testing was not performed in the majority of our patients. When VF testing was performed, it occurred early after presentation and only once. In the patients who underwent VF testing, most of them had some degree of alteration, with most showing advanced stages of the disease. We are not able to explain the specific loss of the superior field in one of these patients. Based on previous reports, the VF mostly shows peripheral commitment when patients are in the advanced stages of the disease and is not correlated with the VA [2, 20, 34, 35].

Information about OCT was retrieved for nine Brazilian patients. The most common alteration was the atrophy of RPE foci. These findings reveal a more advanced stage of the disease, as demonstrated by Skvortsova et al. [26]. Different OCT alterations, not found in our patients, have been described in the literature. Findings such as suprachoroidal hyporeflective spaces seem to be associated with the active leakage phase of the disease. In the same sense, hyperreflective choroidal foci are believed to be active areas of disease, perhaps inflammatory clusters [25–27].

Our study has some weaknesses. One is that the diagnosis and follow-up were performed by different retinal specialists in different years. The data were analyzed and summarized by only one of our researchers. Some of these patients had been lost to follow-up, and the only information available was that in the medical records. These weaknesses led to different treatments being performed and a lack of information on ancillary tests, such as ICG, ERG, and VF testing, in most of our patients. Additionally, the lack of ancillary test information for some patients complicated the grading of ancillary exams such as FA, ICGA and OCT in classification scores, as proposed by Tugal–Tutkun and colleagues for FA [36].

The high heterogeneity of treatments and outcomes did not allow us to reach any conclusions about treatment.

## Conclusion

We report a BRC case series among Brazilian patients. One peculiarity in our sample was the high prevalence of AS inflammation, diverging from the data described in Caucasian patients. Exudative signs were also more prevalent in this series, which could be attributed to seeking assistance in a more symptomatic phase. Another important finding was the advanced stage of the disease at which some patients presented to the ophthalmologist, a finding confirmed by previous studies in Hispanic patients. These data reveal the importance of improving the diagnosis of BRC in Hispanic patients. Another important feature was the high prevalence of “white” individuals in our series. This may suggest a Caucasian ancestry, even though a genetic study would be necessary to confirm this theory.

## Data Availability

The authors confirm that the data supporting the findings of this study are available within the article.

## References

[1] Minos E, Barry RJ, Southworth S, Folkard A, Murray PI, Duker JS (2016). Birdshot chorioretinopathy: current knowledge and new concepts in pathophysiology, diagnosis, monitoring and treatment. Orphanet J Rare Dis.

[2] Priem HA, Oosterhuis JA (1988). Birdshot chorioretinopathy: clinical characteristics and evolution. Br J Ophthalmol.

[3] Rothova A, Berendschot TTJM, Probst K, Van Kooij B, Baarsma GS (2004). Birdshot chorioretinopathy: long-term manifestations and visual prognosis. Ophthalmology.

[4] Herbort CP, Pavésio C, LeHoang P, Bodaghi B, Fardeau C, Kestelyn P (2017). Why birdshot retinochoroiditis should rather be called “HLA-A29 uveitis”?. Br J Ophthalmol.

[5] Ryan SJ, Maumenee AE (1980). Birdshot retinochoroidopathy. Am J Ophthalmol.

[6] Gass JDM (1981). Vitiliginous chorioretinitis. Arch ophthalmol.

[7] Nussenblatt RB, Mittal KK, Ryan S, Green WR, Maumenee AE (1982). Birdshot retinochoroidopathy associated with HLA-A29 antigen and immune responsiveness to retinal s-antigen. Am J Ophthalmol.

[8] Levinson RD, Du Z, Luo L, Monnet D, Tabary T, Brezin AP (2008). Combination of KIR and HLA gene variants augments the risk of developing birdshot chorioretinopathy in HLA-A*29-positive individuals. Genes Immun.

[9] Kuiper J, Rothova A, de Boer J, Radstake T (2015). The immunopathogenesis of birdshot chorioretinopathy; a bird of many feathers. Prog Retin Eye Res.

[10] Levinson RD, Brezin A, Rothova A, Accorinti M, Holland GN (2006). Research criteria for the diagnosis of birdshot chorioretinopathy: results of an international consensus conference. Am J Ophthalmol.

[11] Alves-Silva J, da Silva SM, Guimarães PEM, Ferreira ACS, Bandelt HJ, Pena SDJ (2000). The ancestry of Brazilian mtDNA lineages. Am J Hum Genet.

[12] Pena SDJ, Di Pietro G, Fuchshuber-Moraes M, Genro JP, Hutz MH, Kehdy FdSG (2011). The genomic ancestry of individuals from different geographical regions of Brazil is more uniform than expected. PLoS ONE.

[13] Carvalho-Silva DR, Santos FR, Rocha J, Pena SDJ (2001). The phylogeography of Brazilian Y-chromosome lineages. Am J Hum Genet.

[14] Baddar D, Goldstein DA (2016). HLA-A29-positive Birdshot chorioretinopathy in a hispanic patient. Ocul Immunol Inflamm.

[15] Gasch AT, Smith JA, Whitcup SM (1999). Birdshot retinochoroidopathy. Br J Ophthalmol.

[16] Rodríguez-García A, Almánzar-Santos MA (2012). Coriorretinopatía en perdigonada (Birdshot) en pacientes mexicanos: Espectro clínico y experiencia terapéutica. Rev Mex Oftalmol.

[17] Torres Soriano ME, Moreno G, Jiménez-Sierra JM (2008). Coriorretinopatía en birdshot. Reporte de un caso. Rev Mex Oftalmol.

[18] Gupta V, Sharma VK (2019). Skin typing: Fitzpatrick grading and others. Clin Dermatol.

[19] Fardeau C, Herbort CP, Kullmann N, Quentel G, LeHoang P (1999). Indocyanine green angiography in birdshot chorioretinopathy. Ophthalmol.

[20] Shah KH, Levinson RD, Yu F, Goldhardt R, Gordon LK, Gonzales CR (2005). Birdshot chorioretinopathy. Surv Ophthalmol.

[21] Knecht PB, Papadia M, Herbort CP (2014). Early and sustained treatment modifies the phenotype of birdshot retinochoroiditis. Int Ophthalmol.

[22] Guex-Crosier Y, Pittet N, Herbort CP (1995). Sensitivity of laser flare photometry to monitor inflammation in uveitis of the posterior segment. Ophthalmology.

[23] Guex-Crosier Y, Pittet N, Herbort CP (1994). Evaluation of laser flare-cell photometry in the appraisal and management of intraocular inflammation in uveitis. Ophthalmology.

[24] Vasconcellos AF, Oréfice F, Siqueira RC (1994). Retinocoroidopatia de Birdshot relato de um caso brasileiro Birdshot retinochoroidopathy; a brazilian case report. Rev Bras Oftalmol.

[25] Gonzalez Fernandez D, Nascimento H, Nascimento C, Muccioli C, Belfort R (2017). Uveitis in São Paulo, Brazil: 1053 new patients in 15 months. Ocul Immunol Inflamm.

[26] Thorne JE, Jabs DA, Peters GB, Hair D, Dunn JP, Kempen JH (2005). Birdshot retinochoroidopathy: ocular complications and visual impairment. Am J Ophthalmol.

[27] Llop SM, Papaliodis GN (2018). Cataract surgery complications in uveitis patients: a review article. Semin Ophthalmol.

[28] Lobo AM, Papaliodis GN (2010). Perioperative evaluation and management of cataract surgery in uveitis patients. Int Ophthalmol Clin.

[29] Comander J, Loewenstein J, Sobrin L (2011). Diagnostic testing and disease monitoring in birdshot chorioretinopathy. Sem Ophthalmol.

[30] Sobrin L, Lam BL, Liu M, Feuer WJ, Davis JL (2005). Electroretinographic monitoring in birdshot chorioretinopathy. Am J Ophthalmol.

[31] Wang D, Nair A, Goldberg N, Friedman A, Jabs D, Brodie SE (2020). Oscillatory potentials in patients with birdshot chorioretinopathy. Doc Ophthalmol.

[32] Silpa-Archa S, Lee JJ, Boonsopon S, Lizárraga MT, Preble JM, Sujirarat D (2016). Poor prognostic factors in patients with birdshot retinochoroidopathy. Retina.

[33] Zacks DN, Samson CM, Loewenstein J, Foster CS (2002). Electroretinograms as an indicator of disease activity in birdshot retinochoroidopathy. Graefe’s Arch Clin Exp Ophthalmol.

[34] Priem HA, De Rouck A, De Laey JJ, Bird AC (1988). Electrophysiologic studies in birdshot chorioretinopathy. Am J Ophthalmol.

[35] Vitale AT (2014). Birdshot retinochoroidopathy. J Ophthalmic Vis Res.

[36] Tugal-Tutkun I, Herbort CP, Khairallah M, Allegri P, Biziorek B, Bodaghi B (2010). Scoring of dual fluorescein and ICG inflammatory angiographic signs for the grading of posterior segment inflammation (dual fluorescein and ICG angiographic scoring system for uveitis). Int Ophthalmol.

